# CO_2_ flagging - an improved method for the collection of questing ticks

**DOI:** 10.1186/1756-3305-5-125

**Published:** 2012-06-21

**Authors:** Călin M Gherman, Andrei D Mihalca, Mirabela O Dumitrache, Adriana Györke, Ioan Oroian, Mignon Sandor, Vasile Cozma

**Affiliations:** 1 Department of Parasitology and Parasitic Diseases, University of Agricultural Sciences and Veterinary Medicine Cluj-Napoca, Calea Mănăştur 3-5, Cluj-Napoca 400372, Romania; 2 Department of Plant and Environmental Protection, University of Agricultural Sciences and Veterinary Medicine Cluj-Napoca, Calea Mănăştur 3-5, Cluj-Napoca 400372, Romania

**Keywords:** Flagging, Carbon dioxide, Questing ticks, *Ixodes ricinus*

## Abstract

**Background:**

Most epidemiological studies on tick-borne pathogens involve collection of ticks from the environment. An efficient collection method is essential for large sample pools. Our main aim was to evaluate the efficacy of a new method, where traditional flagging was enhanced by the use of CO_2_ dispersed into the white flannel. The CO_2_ was spread through a rubber hose network inserted into the flag blanket. The research was conducted in spring, in March-April 2011 in two locations from Cluj County, Romania.

**Methods:**

The research was conducted in March-April 2011 in two locations from Cluj County, Romania. The flag to be tested contained a fine silicone rubber hose network which dispersed the CO_2_ in the shaft. On each collection site n=30 samplings were performed. Each sampling consisted in the simultaneous use of both flags (with and without CO_2_) by two persons. The CO_2_ concentration level on the flag canvas surface was measured. The efficacy of the method was determined by counting comparatively the total number of ticks and separate developmental stage count.

**Results:**

Using the CO_2_ improved flag, 2411 (59%) *Ixodes ricinus* and 100 (53.8%) *Dermacentor marginatus* ticks were captured, while the CO_2_-free flag accounted for the collection of 1670 *I. ricinus* (41%) and 86 (46.2%) *D. marginatus* ticks. The addition of CO_2_ prompted a concentration difference on the surface of the flag ranging between 756.5 and 1135.0 ppm with a mean value of 848.9 ppm.

**Conclusion:**

The study showed that the CO_2_ enhanced sweep flag increased the ability of *I. ricinus* (p < 0001) but not of *D. marginatus* to be attracted to the flag blanket.

## Background

Ticks (suborder Ixodida) are obligate blood-sucking acarines attacking a wide variety of hosts from all tetrapod vertebrate classes [1,2]. Around 700 species of hard ticks are currently recognized as valid species [1]. Most of these species are three-host ticks (i.e. each stage detaches after engorgement) [3,4]. Regardless of the number of hosts, each tick must find a suitable host. In three-host ticks, most of their multiannual life is not spent attached to the host but as free-living organisms. Thus, newly hatched larvae, unfed nymphs and unfed adults are in a permanent host finding state. Host detection and attachment in Ixodidae is achieved through three main alternative behavioral patterns: questing, hunting and tick-host cohabitation (nidiculous ticks) [4].

Most epidemiological studies on tick-borne pathogens involve collection of ticks from the environment [5]. Thus, an efficient collection method is essential for large datasets. Tick collection methods had been reviewed by Gray [6]. He divided these methods into four major categories: (1) flagging or dragging methods; (2) trapping using carbon dioxide baits; (3) collecting from hosts and (4) walking (i.e. on the clothes of the collectors). Despite all these methods are relative and do not estimate density (number per unit area) or absolute size (total number as measured in mark-release-recapture methods) [7], each of these has a variable efficacy depending on several factors (i.e. habitat type, tick species, developmental stage etc.). Nevertheless, all methods have been improved over the time in order to increase their efficacy [8].

One of the most important ticks species (regarding its range, abundance, and vectorial importance) in the Palearctic region, with tendency to expand its spread in Northern Europe [9,10], is *I*x*odes ricinus*[11]. The host seeking strategy of all developmental stages in *I. ricinus* is questing, when ticks are typically positioned on the vegetation with their legs extended, waiting for a moving host to which they attach [12]. Though, questing is a complex behavioral process, which involves responses to stimuli like host movement, concentration of environmental carbon dioxide and increase of temperature [4]. The most commonly used method for collection of questing ticks is flagging. However, flagging stimulates only the tick sensor for movement, and leaves the other two sensorial components of questing (i.e. carbon dioxide and temperature) unexploited.

The vast majority of ecological and epidemiological studies of tick-borne pathogens involve collection of unfed ticks from the environment. In this view, our main aim was to evaluate the efficacy of a new method, where traditional flagging was enhanced by the use of dispersed CO_2_ into the white flannel.

## Methods

### Sweep and flag design

**Figure 1:**
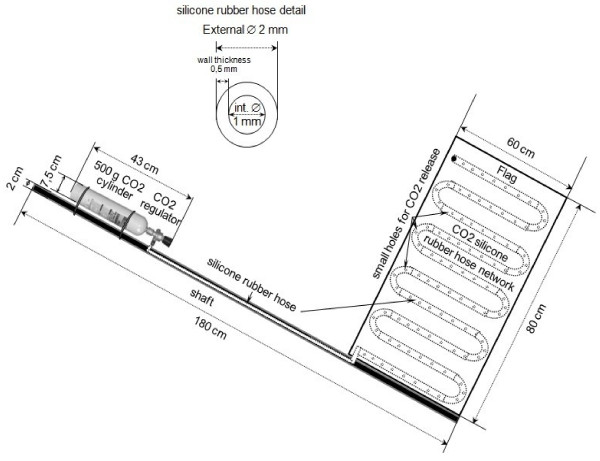
**Sweep CO**_**2**_**flag diagram.**

The sweep consists of a shaft and a flag. The shaft is constructed from a hollow aluminum tube, and the flag from white technical flannel. A JBL 500 g CO_2_ bottle with a CO_2_ solenoid regulator attached (both aquarium use, JBL Aquarium®, Germany) were fixed with plastic lock seals on the shaft. A silicone rubber hose was attached to the CO_2_ solenoid regulator; the hose was introduced through the aluminum shaft and connected to the flag. The rubber hose was pierced (to release CO_2_) and attached to the flag by sewing forming a network structure (Figure 1). The hose was made of bending-resistant silicone rubber with the inner diameter of 1 mm and the outer diameter of 2 mm with a wall thickness of 0.5 mm.

The flag surface area was 0.48 m^2^ (80 x 60 cm) to allow unrestricted passage across all types of vegetation. Two identical flags were made; one of them with CO_2_ and the other without CO_2_ (control).

### Study area

**Figure 2:**
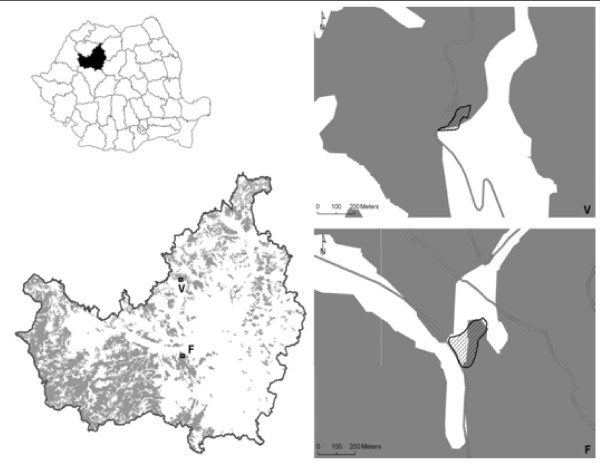
Field studies location (shaded area shows the flagging surface).

Two hilly areas were chosen: Vultureni and Faget, both in Cluj county (Figure 2), according to preliminary results of sampling for the evaluation of the presence of tick-borne pathogens in Romania (manuscript under preparation). The habitats consisted in herbaceous vegetation alternating with small shrubs, located at the edge of woods, specific areas for ticks. The climate is moderate continental, influenced by the vicinity of the Apuseni Mountains and Atlantic influences from west of the country, in autumn and winter [13]. The study was conducted in spring, between the end of March and the end of April 2011, as tick abundance is higher in Northwestern Romania in this season [14]. The GPS was used to measure the distance.

### Sampling procedure

On each collection site n = 30 samplings were performed (total 60 samplings in the two sites). Each sampling consisted in the simultaneous use of both flags, (with and without CO_2_), on 2 m wide adjacent areas, by two persons who had interchanged the sweep every fifty meters for the homogeneity of results. After each 5 m the flags were checked for ticks. All ticks were collected regardless their species and fixed in pure ethanol. Specific identification was performed using morphological keys [15] under a binocular microscope.

### Determination of carbon dioxide

The CO_2_ concentration level on the flag canvas surface was measured using a portable CIRAS-2 Photosynthesis System equipped with a SRC-1 Soil Respiration Chamber (PP Systems International Inc®, USA). CO_2_ level was determined at a single time, but from several points of the flag (n = 20) according to instruction manual and recommendations [16]. The results were expressed in ppm as compared to the standard CO_2_ concentration of 393.71 ppm (reference data for 24.04.2011) [16].

### Statistical analysis

The efficacy of the method was determined by counting the total number of ticks and separate developmental stage count, attached to the CO_2_ flag compared with the control (CO_2_-free flag). For statistical analysis, the values were compared in CHI-SQUARE TEST [17]. A p value of <0.05 was considered statistically significant. The relative risk (RR) is a ratio of the probability of the event occurring in the exposed group versus a non-exposed group [18].

## Results

A total number of 4267 of ticks belonging to two species were collected in the 60 samplings: *I. ricinus* (n = 4081) and *Dermacentor marginatus* (n = 186).

*I. ricinus* accounted for 4081 tick captures (adults, nymphs and larvae) were collected in the 60 samplings (Table1). Of these, 2617 (64.1%) were adults, 1422 (34.9%) nymphs and 42 (1%) larvae. Using CO_2_ improved flag were captured 2411 (59%) ticks and 1670 (41%) without CO_2_.

**Table 1:** **Number of questing** ***Ixodes ricinus*** **ticks collected by flagging with and without CO**_**2**_

**Location/stage/method**		**Faget**		**Vultureni**		**Total (both locations)**	
		IR	DM	IR	DM	IR	DM
**Males**	CO_2_+	456	27	211	22	667	49
	CO_2_-	325	21	160	20	485	41
**Females**	CO_2_+	587	26	281	25	868	51
	CO_2_-	407	23	190	22	597	45
**Nymphs**	CO_2_+	547	0	304	0	851	0
	CO_2_-	353	0	218	0	571	0
**Larvae**	CO_2_+	22	0	3	0	25	0
	CO_2_-	16	0	1	0	17	0
**TOTAL**	CO_2_+	1612	53	799	47	2411	100
	CO_2_-	1101	44	569	42	1670	86
	Per location	2713	97	1368	89	4081	186

The statistical analysis revealed highly statistically significant (p < 0001) difference between the two variables in adults and nymphs, in both locations and overall; for larvae, the recorded statistical differences were not significant (Table2). Carbon dioxide flagging was more effective than CO_2_-free flagging, with average values of RR ranging between 1.4 and 1.6 for adults and nymphs.

**Table 2:** **Statistical significance of tick collection efficacy using flagging with and without CO**_**2**_

**Location**		**Faget**		**Vultureni**		**Total**	
**Statistical parameter**		***I. ricinus***	***D. marginatus***	***I. ricinus***	***D. marginatus***	***I. ricinus***	***D. marginatus***
		**P**	**P**	**P**	**P**	**P**	**P**
		**RR**	**RR**	**RR**	**RR**	**RR**	**RR**
**Males**	**CO**_**2**_**+**	0.0001	0.31	0.0002	0.83	0.0001	0.30
		1.40(1.27–1.56)	1.29(0.86–1.91)	1.32(1.14–1.53)	1.1(0.72–1.69)	1.38(1.27–1.50)	1.20(0.89–1.61)
	**CO**_**2**_**-**						
**Females**	**CO**_**2**_**+**	0.0001	0.69	0.0001	0.68	0.0001	0.47
		1.44	1.13(0.76–1.68)	1.48(1.30–1.67)	1.14(0.76–1.71)	1.45(1.35–1.57)	1.13(0.85–1.51)
	**CO**_**2**_**-**	(1.32–1.58)					
**Nymphs**	**CO**_**2**_**+**	0.0001		0.0001		0.0001	
						1.5 (1.4–1.6)	
	**CO**_**2**_**-**	1.6 (1.4–1.7)		1.4 (1.2–1.6)			
**Larvae**	**CO**_**2**_**+**	0.25		0.48		0.13	
	**CO**_**2**_**-**	1.4 (0.9–2.2)		3 (0.5–18)		1.5 (0.9–2.3)	

The addition of CO_2_ prompted a difference on the surface of the flag ranging between 756.5 and 1135.0 ppm with a mean value of 848.9 ppm.

## Discussion

Flagging is the most widespread tick collection method. Over time, several improvements to this method were proposed [8] and now there is many types used: double walking flagging and double walking with baited flagging [19], walking flagging with a loose-fitting and white cotton flannel garment worn [20] and strip-flag method [21].

The successful hosts attack in *Ixodes* species expressed as the ability to adhere to a flannel flag is influenced by many factors: light and/or shadow, radiation heat (temperature), mechanical vibration of questing substrate, host odor, and CO_2_ concentration [22].

Chemical mediators also known as semiochemicals are as well important for behavioral patterns in ticks. These information-bearing compounds are secreted by animals into the external environment, and when recognized they trigger a specific behavioral response such as food location, sexual partner location or escape [23]. Semiochemicals are categorized into four major categories: (1) pheromones; (2) allomones; (3) kairomones; and (4) synomones [24].

Carbon dioxide acts like an attractant kairomone for ticks [25]. Experimentally, it acted as an attracting agent causing almost immediate activation in the soft ticks *Ornithodoros coriaceus* quiescent ticks [25]. Adults of *Dermacentor andersoni* respond also very well to stimulation with carbon dioxide. Garcia, 1965, described a system based on release of CO_2_ for the collection of *D. andersoni*. The system involved a piece of dry ice placed on a wire mesh platform in the desired area. The results indicate that the CO_2_ method is more sensitive for detection of adult ticks than is the conventional flagging technique [26]. Carbon dioxide (dry ice) trapping method was demonstrated to be effective in the collection of some tick species: *I. ricinus*[5], *Amblyomma americanum*[27-29] and *A. hebraeum*[30]. Our method is important in Europe as it enhances the capture of *I. ricinus*, the main vector of *Borrelia burgdorferi* s.l. and the tick-borne encephalitis (TBE) virus [31].

Our results are consistent with those cited above. The number of adults and nymphs of *I. ricinus* collected was significantly increased using CO_2_ enhanced blanket comparing with flagging without CO_2_. The lower number of larvae collected can be explained by the months of sampling, March-April, when larvae may be not fully active and by the quality of blanket, made by technical flannel, material which may not have reached the lower levels of the vegetation where larvae sit to quest.

These data show that the responsiveness to CO_2_ is enhanced during host-seeking periods of the life cycle and reduced at other times [32]. However, others [29] did not establish significant differences between flagging method with a strip blanket and CO_2_ traps or rabbits scent baits. The response time of ticks to host attachment was shown to be dependent on the tick species and CO_2_ concentration in the environment [33]. In *Amblyomma maculatumA. americanum* and *Dermacentor variabilis*, the groups preconditioned with low ambient CO_2_ (422 ppm) always produced response times of longer duration than ticks preconditioned to high ambient CO_2_ (956 ppm) [34].

The lack of statistical significance for *D. marginatus* between the two collecting methods compared in the present study might be caused by the major differences in the sample size. The small total number of *D. marginatus* collected (regardless the method used) can be explained by the typical area of this species which prefers biotopes characterized by xerophilic plant communities: dry pasture shrub communities, grazing black locust forests (*Robinietum*), forest-steppe or grikes (*Quercetum pubescentis* and *CometoQuercetum*), margins of oak forests and bushy ridges between the fields and field paths [35]. Our study area is characterized by deforested hills and covered with low vegetation; predominant species in forest areas are hornbeam, birch, poplar, hazel, elm, ash and maple. Although *D. marginatus* is almost as widespread in Romania as *I. ricinus*, [36], the population density is significantly lower in most of the sampled localities [37]. Regarding seasonality, in Eastern Europe, *D. marginatus* is most numerous in February and March [38] and most of our sampling was done in April.

Flagging technique seems to work better for other species: *Ixodes dammini*[27], *I. pacificusD. occidentalis* and *D. variabilis*[32], *I. rubicundus*[39] or *I. ricinus*[40]. Four different methods of surveying were tested for *D. variabilis* and *I. banksi* and it was shown that the most successful was flagging, compared with carbon dioxide trapping, nest boxes and collection from hosts [41].

Concerning the temperature, *I. persulcatus* is a more cold-resistant tick than *I. ricinus* and it is more successful both in adhering to the flag and in remaining attached to it at two ranges: 6–10°C and 17–22°C [42]. In general a greater percentage of *I. rubicundus* displayed an appetence response at lower (12, 17 and 21°C) than at high (30°C) temperatures [39]. It is known that all life stages of *I. ricinus* are equipped to sense shifts in light intensity. This allows *I. ricinus* to use onset of darkness to trigger mobility when desiccation risk is reduced in nature [43]. The nymphs are stimulated to walk horizontally by humidity and host scent. When the atmosphere is sufficiently wet they are likely to walk towards odor secreted by host skin [44,45]. The larvae of *I. hirsti* seem to be more sensitive to shade and heat, while they were unresponsive to CO_2_ concentration and host odor [46]. Radiation heat and shadowing caused the greatest percentage of *I. rubicundus* to display an appetence response; shadowing and radiation heat had the least effect on *R. punctatus*[22]. A single mechanical perturbation of the substratum caused a mean of 50% of *I. rubicundus* to display an appetence response. Constant mechanical perturbation resulted in a progressive decrease in the proportion of ticks reacting [22]. Host scent is known to initiate questing behavior in *I. persulcatusI. ricinus* and *I. crenulatus*. Both *I. ricinus* and *I. crenulatus* respond strongly to sheep wool [47,48].

Entire-blanket flagging is a better sampling method for *I. ricinus* comparing with others variants of flagging or dragging. Significantly more nymphs and adults were caught by the entire-blanket versus strip-blanket flagging [49]. Flagging was 1.5–1.7 times as effective as dragging; impregnation of the cloths with different substances, like host odor, increased the efficacy by 2.4 (dragging) to 2.8 (flagging) times [39]. From several types of material used (i.e. cotton, woolen flannel, “molleton” - soft thick cotton, and toweling - spongecloth), the last was the best cloth type to optimize the number of ticks collected [36]. However, dry-ice-baited tick-traps is more effective for ticks with increased mobility, like *A. americanum*[27].

Our enhanced technique that combines, for the first time, classical flagging and carbon dioxide trapping methods improved significantly the ability of *I. ricinus* to adhere to flag blanket. By introducing CO_2_ in the white blanket we tried to simulate the time approach of a host to the tick. Sudden increase of the CO_2_ concentration in the air stimulates its vivacity and questing position, increasing the chance of attachment of ticks to the flag.

## Conclusion

The study showed that the CO_2_ enhanced sweep flag increased the ability of *I. ricinus* (p < 0001) but not of *D. marginatus* to be attracted to the flag blanket.

## Competing interests

The authors declare that they have no competing interests.

## Authors’ contributions

GCM wrote the manuscript, performed flagging. MAD concept idea, performed the flagging. DMO identified the ticks. GA statistical analysis. OI measurement of CO_2_. SM measurement of CO_2_. CV team coordinator. All authors read and approved the final manuscript.
